# COVID-19 infection control education for medical students undergoing clinical clerkship: a mixed-method approach

**DOI:** 10.1186/s12909-022-03525-1

**Published:** 2022-06-12

**Authors:** Hajime Kasai, Go Saito, Shoichi Ito, Ayaka Kuriyama, Chiaki Kawame, Kiyoshi Shikino, Kenichiro Takeda, Misuzu Yahaba, Toshibumi Taniguchi, Hidetoshi Igari, Seiichiro Sakao, Takuji Suzuki

**Affiliations:** 1 Health Professional Development Center, Chiba University Hospital, Chiba, Japan; 2 Department of Respirology, Graduate School of Medicine, Chiba University, Chiba, Japan; 3 Department of Medical Education, Graduate School of Medicine, Chiba University, Chiba, Japan; 4 Department of General Medicine, Graduate School of Medicine, Chiba University, Chiba, Japan; 5 Department of Infectious Diseases, Chiba University Hospital, Chiba, Japan

**Keywords:** COVID-19, Role-playing, Simulation training, Lecture, Qualitative research, Focus groups

## Abstract

**Background:**

Coronavirus disease (COVID-19) has induced an urgent need to train medical students not only in infection prevention control but also in the treatment of infectious diseases, including COVID-19. This study evaluates the impact of simulated clinical practice with peer role-plays and a lecture on clinical education for COVID-19.

**Methods:**

The sample for the study included 82 fourth- and fifth-year medical students undergoing clinical clerkship in respiratory medicine. They answered questionnaires and participated in semi-structured focus group interviews (FGIs) regarding the advantages of simulated clinical practice with peer role-plays and lectures on clinical education for COVID-19.

**Results:**

A total of 75 students participated in the COVID-19 education program between January and November 2021. The responses to the questionnaire revealed that the satisfaction level of students with COVID-19 education was high. No significant change was found among students concerning fear of COVID-19 before and after the program. The degree of burden of handling information on COVID-19 reduced significantly, while the degree with respect to the use of personal protective equipment (PPE), including appropriate wearing and removing of PPE, and care of patients with confirmed COVID-19 while taking steps to prevent infection, exhibited a decreasing trend. Nine FGIs were conducted (*n* = 74). The advantages of simulated clinical practice were segregated into five categories (infection prevention control, educational methods, burden on healthcare providers, self-reflection, and fear of COVID-19); and that of the lecture were segregated into four categories (information literacy, knowledge of COVID-19, educational methods, and self-reflection).

**Conclusions:**

Simulated clinical practice with peer role-plays and the lecture pertaining to COVID-19 can prove to be efficient and safe methods for learning about COVID-19 infection and prevention control for medical students. They can reduce the burden of COVID-19 patients’ care. Moreover, they can also provide an opportunity for self-reflection, realize the burden of medical care, and acquire relevant information.

## Background

Since February 2020, coronavirus disease (COVID-19) has spread rapidly worldwide. One of the many ways this pandemic has affected society is its impact on medical education [1, 2]. There is an urgent need to prevent COVID-19 infection among medical students and train medical personnel to manage infectious diseases, including COVID-19. However, it is necessary to develop and apply appropriate educational methods for infection prevention control (IPC) that can be used in clinical settings. Such educational methods have not been established thus far.

Simulation-based education enables safe and effective iterative learning [3, 4]. Simulation and online learning played a major role in the early phase of COVID-19 in China [5]. Peer role-play is also a low-cost method that can be integrated into practice [6]. Some reports on educational practices focusing on IPC for COVID-19 have been made available by medical professionals working in the field and by nursing students [7–9]. However, there are few reports of educational practices on IPC for COVID-19 among medical students. Additionally, IPC for COVID-19 can be imparted to medical students as part of actual medical practice.

In March 2020, the clinical clerkship (CC) at our institution was suspended due to COVID-19. In July, a questionnaire survey of medical students before their CC resumed revealed that some of them were afraid to continue with their CC. Although our institution had treated many cases of COVID-19, medical students were not provided adequate information about the actual COVID-19 situation in our institution. In addition, they were likely to find it difficult to identify relevant details on COVID-19 from the plethora of information (i.e., infodemic) available. Consequently, medical students, who were still in the developmental stage as medical professionals, felt a vague sense of anxiety and fear in relation to this unknown infectious disease. In fact, in the United States, most medical students felt somewhat anxious about the risk of being infected with COVID-19 [10]. Therefore, in addition to the implication of safe CC, it was necessary to develop educational methods for CC that would allow medical students to learn appropriate IPC, reduce their anxiety, and feel less burdened due to COVID-19 [11].

We conducted a simulated clinical practice exercise for dealing with COVID-19 using peer role-plays. A lecture was also conducted to provide accurate information about COVID-19 and remind the students to be careful in handling the information. We evaluated the effect of our program on students’ responses to COVID-19 with a mixed-method approach that incorporated quantitative and qualitative techniques [12–14]. The quantitative phase comprised a questionnaire-based research design, while the qualitative phase was based on focus group interviews (FGIs).

## Methods

### Aim

This study evaluates the effects of simulated clinical practice using peer role-plays and a lecture on COVID-19 on medical students’ attitudes to COVID-19 and the burden felt by them due to COVID-19 patients’ care.

### Ethical approval

This study was approved by the Ethics Committee of Chiba University (approval no. 3425). The study database was anonymized.

### Setting

#### CC in the department of respiratory medicine and participants

Medical schools in Japan offer a six-year curriculum, and two years are generally spent in CCs [15]. In Chiba University, with approximately 120 students in each class, students practice in one department and then in another on a rotational basis, every four weeks for two years. The CC begins in December of the fourth year and ends in October of the sixth year.

Groups of 7–11 medical students (4th–5th year) underwent four weeks of training as members of a medical team of doctors and residents in a department of respiratory medicine from December of their fourth year to November of their fifth year. A total of 82 medical students underwent CC in respiratory medicine at Chiba University Hospital between December 2020 and November 2021. During the orientation at the beginning of each group’s CC, informed consent was obtained from the participants for the use of their data for this study.

Between January 2020 and November 2021, students who participated in both the simulated clinical practice and the lecture were included in the study. Students who did not participate in either the simulated clinical practice and/or the lecture, or those with insufficient data from the questionnaire were excluded. Futhermore, medical students did not directly examine patients with COVID-19; rather, they only conducted telephonic interviews and shared information at conferences when they were in charge of these patients. However, the seven students who practiced in September 2021, during the fifth wave of the pandemic in Japan, performed a direct examination of the patients after the simulated clinical practice for COVID-19. Therefore, these seven students were also excluded from the study because of the possible impact of the learning effect of directly participating in the care of patients with COVID-19.

#### Simulated clinical practice for COVID-19 using peer role-plays

The clinical practices were conducted at the simulation center (Chiba Clinical Skills Center) in the Chiba University Hospital and included seven to eight medical students in the first or second week of the CC. Two of the authors (HK and AK) supervised the practice.

**Fig. 1:**
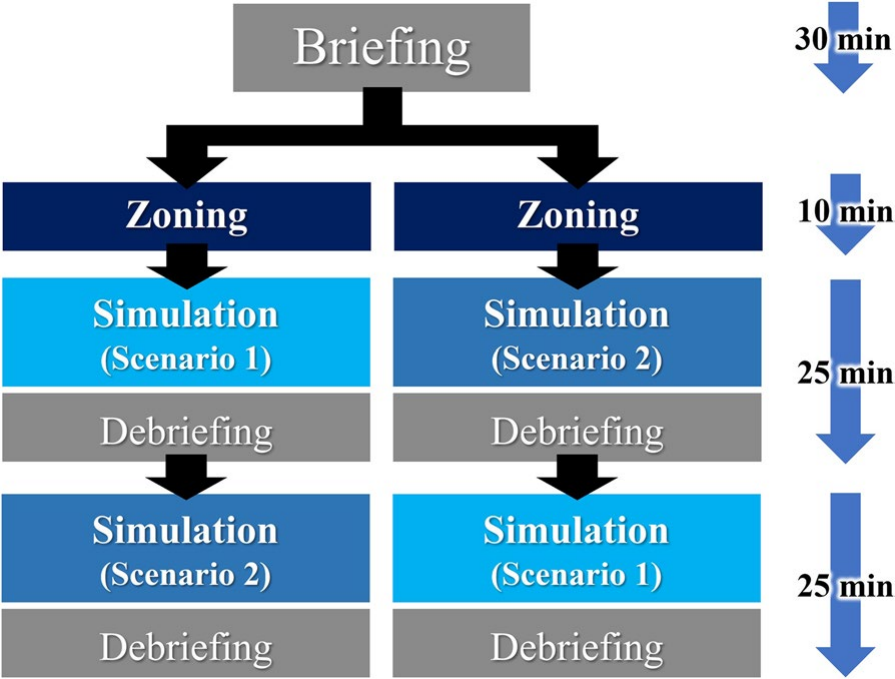
The process of simulated clinical practice involving peer role-play

Before the practice, students were briefed about basic IPC, such as wearing and removing PPE and zoning (Fig. 1); they also attended an orientation session.

Students were divided into two groups of four. After their zoning practice of the simulation center, further practice was conducted based on different scenarios pertaining to the admission of patients with COVID-19. We prepared two scenarios with different patient settings and lead lines for the hospital room, and each group participated in the practice according to the two scenarios.

**Fig. 2:**
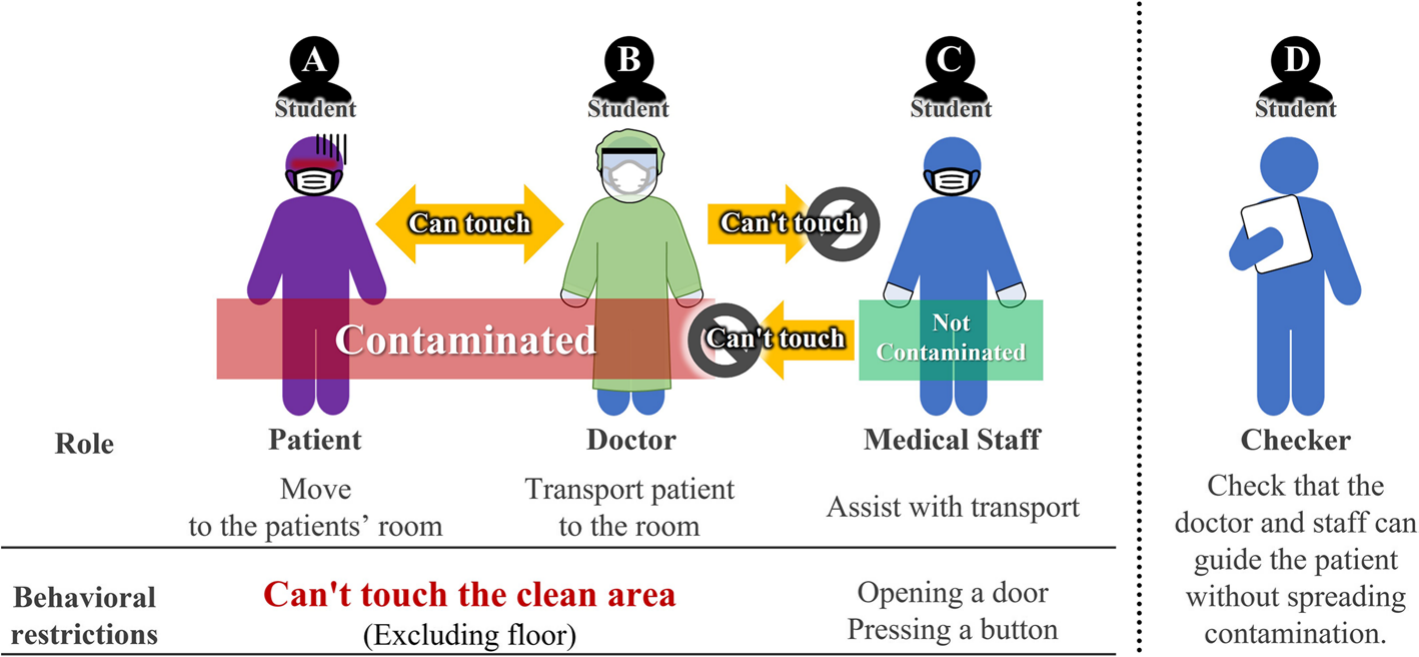
The role settings in the simulated clinical practice during peer role-play

**Fig. 3:**
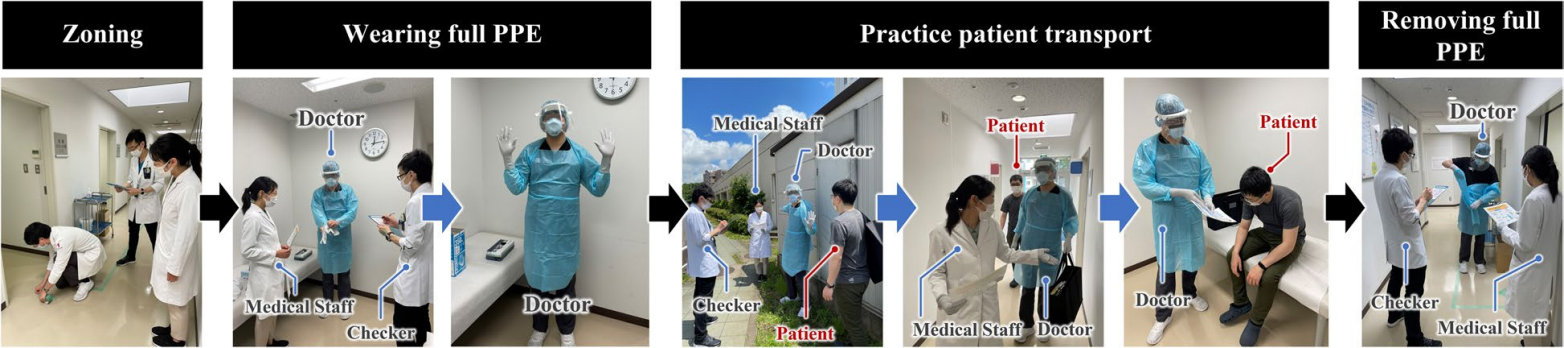
Flow of guiding a COVID-19 patient to the patient room and tasks for each role in the simulated clinical practice with peer role-play using the photographs reproduced by the authors and staff of the Department of Respiratory Medicine. COVID-19: Coronavirus disease 2019; PPE: personal protective equipment

For each scenario, four students were assigned the following roles: a patient, a doctor who wore full PPE, a medical staff who assisted the doctor, and a checker who checked the doctor and the medical staff (Fig. 2). In the role-play, the patient and the doctor could touch each other, but they were considered contaminated and could not touch the clean area and the medical staff. The medical staff could touch the clean area (open the door, push a button, etc.) to maintain cleanliness, while they could not touch the patient and the doctor. As a scenario, we created a fictional patient setting based on an actual acceptance form and prepared a script for each role. During practice, students who played the role of the doctor and the medical staff were supposed to practice how to admit the patient to the ward appropriately without spreading the infection (Fig. 3).

During the debriefing after the practice, the checkers reported the problems, and the students who performed the roles shared their impressions.

#### Lecture on COVID-19

A lecture on the latest literature available on COVID-19 was delivered to students. It included the following themes: comparison of symptoms/problems associated with COVID-19 and influenza, severe acute respiratory syndrome, middle-east respiratory syndrome, clinical findings and treatment of COVID-19, SARS-CoV-2 vaccine, and ways to deal with information regarding COVID-19. Additionally, the lecture included information literacy as follows. We first introduced the research data showing that false news—which evokes fear, disgust, and surprise—is more likely to spread and did so even before the pandemic [16]. Following this, we provided examples of information that later turned out to be untrue, ranging from rumor-level information to those presented by medical professionals and heads of state. We also pointed out that drugs that show promise in basic research are rarely truly useful and approved by regulatory authorities [17]. These examples emphasized the difficulty of properly handling medical information from any standpoint and revealed the process of medical validation using several drugs/vaccines [18]. The lecture highlighted the importance of not easily trusting information unless medical students experience it first-hand.

The lecture was delivered during the third week of the CC by two authors of this paper (GS, HK).

### Data collection

#### Quantitative data collection

Quantitative data were compiled using a questionnaire to evaluate the effect of the education program on students’ responses to COVID-19. Questionnaires were created on students’ fear of COVID-19 and their burden in various situations related to COVID-19 patient care. Before the simulated practice and on the last day of the third week of the CC, students responded to the following questionnaire items on the simulated clinical practice and lectures (Table 1): (1a) Are you afraid of COVID-19? (1b) How much care do you take in your daily life to prevent COVID-19? (2) How much of a burden do you consider the following behaviors? Questions (1a) and (1b) were scored on a five-point Likert scale, with scores ranging from 1 [(1a) Not afraid at all; (2b) Not at all cautious] to 5 [(1a) Very afraid; (1b) Very cautious]. In question 2, the following actions are listed: a. Implementation of COVID-19 prevention measures (daily life), b. General practice while taking COVID-19 preventive measures, c. Appropriate use of PPE, including donning and doffing, d. Care of confirmed COVID-19 patients and adoption of preventive measures, e. Handling of information on COVID-19. Additionally, question (2) was scored on a five-point Likert scale, with scores ranging from 1 (Not burdened at all) to 5 (Very burdened). In addition to the above questions, students reported their satisfaction level with the simulated clinical practice and the lecture on the second questionnaire. The questionnaire items were developed based on the students’ sense of burden in the medical treatment assumed from the teaching process.

**Table 1:** Questionnaire for assessment of students’ awareness and the burden felt due to COVID-19

Question
**1. Awareness**
a. Are you afraid of COVID-19? (Responses were obtained on a 5-point Likert scale with responses ranging from “not afraid at all [1 point], to “very afraid” [5 points])
b. How much care do you take in your daily life to prevent COVID-19? (Responses were obtained on a 5-point Likert scale with responses ranging from “Not at all cautious” [1 point] to “Very cautious” [5 points])
**2. Burden**
Are you afraid of COVID-19?
a. Implementation of COVID-19 prevention measures (daily life)
b. General practice while taking COVID-19 preventive measures
c. Appropriate use of PPE, including donning and doffing
d. Care of confirmed COVID-19 patients and adoption of preventive measures
e. Handling of information on COVID-19 (Responses were obtained on a 5-point Likert scale with responses ranging from “Not burdened at all” [1point]; “Very burdened” [5 points])

*COVID-19* Coronavirus disease 2019

#### Qualitative data collection

We conducted focus group interviews (FGIs) with the students to evaluate the effects and advantages of our program on COVID-19. The FGI also aimed to identify what the students learned through our program. On the last day of the third week, students participated in the semi-structured FGIs regarding the advantages of the program, and this qualitative study phase helped us explain the results of the quantitative data.

The students were divided into nine groups (75 student cohorts in total). The criteria for selection specified that all medical students were to be included, as the target population had to be homogeneous to investigate perceptions regarding our education of COVID-19.

FGIs were conducted by two physician researchers (HK and GS), and the interview responses were recorded independently using an interview guide (Table 2). Students were asked the following questions: *1) “What are the advantages of the simulated clinical practice with peer role-playing about COVD-19? Why do you consider these as advantages?” 2) “What are the advantages of the lecture about COVID-19? Why do you consider these as advantages?”* The interview guide was validated by the two researchers (HK and GS) before data collection.

**Table 2:** Interview guide of the focus group interview

Introductory conversation	Thanking the participants Statement of the aim of the study Procuring oral informed consent Seeking permission to record the conversation on audiotape
Flow of the interview: Statements and questions (Note: care should be taken to respect the flow of discussion)	a.Please introduce yourself
	a.What are the advantages of the simulated clinical practice with peer role-playing about COVID-19? Why do you consider these to be advantages?
	a.What are the disadvantages of the lecture about COVID-19? Why do you consider these to be disadvantages?
Conclusion	Would anybody like to say anything else about this topic?

*COVID-19* Coronavirus disease 2019

The interviews took no longer than 30 min and information on the work impact and fatigue in interviewees were obtained. The interview responses were transcribed verbatim.

### Data analysis

#### Statistical analysis

The quantitative data are expressed as mean ± standard deviation (SD) unless otherwise indicated. The Wilcoxon signed-rank test was used to compare the degree of burden before and after our education regarding COVID-19. Statistical significance was set at *p* < 0.05. All statistical analyses were performed using JMP 16.0 (Cary, North Carolina, USA).

#### Qualitative content analysis

In line with previous studies, qualitative content analysis was performed to analyze the FGI transcripts [19]. Such an analysis comprises descriptions of the manifested content and interpretations of the latent content [20]. HK and CK independently read and coded all the transcripts. Subsequently, they discussed, identified, and agreed on the coding of the descriptors. Inter-rater reliability was measured with the Kappa coefficient (0.8–1.0 = almost perfect; 0.6–0.8 = substantial; 0.4–0.6 = moderate; 0.2–0.4 = fair) [21].

## Results

A total of 83 medical students participated in the simulated clinical practice for COVID-19 using peer role-plays and the lecture. In total, 82 students completed the questionnaire.

The seven students who had practiced in September 2021 were excluded, and 75 students were finally included in the study. Additionally, nine semi-structured FGIs were conducted (*n* = 74).

### Questionnaire results

**Fig. 4:**
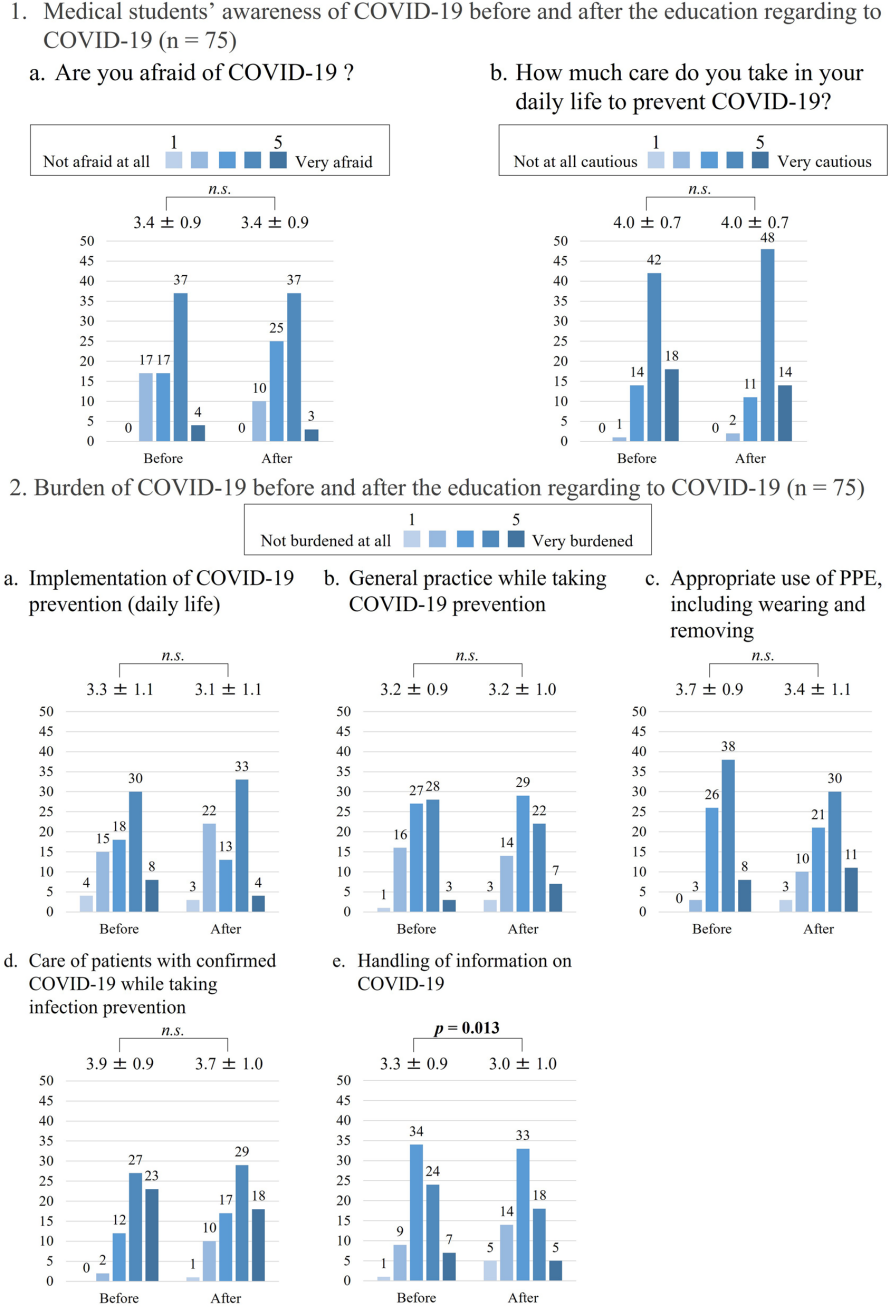
Comparison of the students’ awareness and burden felt sue to COVID-19 before and after the simulated practice involving peer role-play and lecture (n = 75). *n.s*: not significant**.** COVID-19: Coronavirus disease 2019; PPE: personal protective equipment

The responses to the questionnaire indicated that the students’ satisfaction level with the simulated clinical practice and the lecture was high (simulated clinical practice, 4.7 ± 0.5; lecture 4.9 ± 0.3). Figure 4 summarizes the students’ awareness and assumption of the burden of COVID-19 before and after the education program regarding COVID-19 (*n* = 75). There was no change in the degree of fear pertaining to COVID-19 and attention to infection prevention in daily life (from 3.4 ± 0.9 to 3.4 ± 0.9, *p* = 0.462; from 4.0 ± 0.7 to 4.0 ± 0.7, *p* = 0.772, respectively). The burden of handling information on COVID-19 decreased significantly after the program (from 3.3 ± 0.9 to 3.0 ± 1.0, *p* = 0.013). The burden regarding the appropriate use of PPE, including wearing and removing it, and care of those with COVID-19 while taking necessary precautions for prevention of infection decreased (from 3.7 ± 0.9 to 3.4 ± 1.1, *p* = 0.125; from 3.9 ± 0.9 to 3.7 ± 1.0, *p* = 0.095, respectively). In contrast, there was no change in the burden of implementation of COVID-19 prevention (daily life) and general practice while taking measures for COVID-19 prevention (from 3.3 ± 1.1 to 3.1 ± 1.0, *p* = 0.816; from 3.2 ± 0.9 to 3.2 ± 1.0, *p* = 1.000, respectively).

### FGI results

The advantages of the simulated clinical practice were segregated into five categories (infection prevention control, educational methods, burden on healthcare providers, self-reflection, and fear of COVID-19); and that of the lecture were segregated into four categories (information literacy, knowledge of COVID-19, educational methods, and self-reflection). Simulated clinical practice and the lecture included information on 22 and 26 concepts, respectively (Table 3).

**Table 3:** Advantages of the simulated clinical practice involving peer role-play and a lecture on COVID-19 (*n* = 74)

Categories	Concepts	Frequency of each concept	Total
**Simulated clinical practice with peer role-play about COVID-19**			
Infection prevention control	Understanding the process of wearing and removing PPE	36	82
	Understanding zoning	15	
	Importance of infection prevention control	10	
	Management of infected patients on admission	9	
	Knowledge of N95 masks	7	
	Handling of contaminated materials	3	
	Importance of informing the public	1	
	Understanding the risk of infecting oneself	1	
Educational methods	Satisfaction with the simulation	20	45
	Contents useful for the future	8	
	Understanding of the importance of collaboration among medical proffessions	6	
	Role-play/practicing actual medical management	5	
	Learning from various perspectives of medical proffessions	3	
	New awareness through experience	2	
	Sense of security through simulation	1	
Burden on healthcare providers	Understanding the burden on the healthcare providers	12	18
	Understanding the difficulty faced while dealing with the patients	5	
	Realizing the need for medical resources	1	
Self-reflection	Reflecting on practice and understanding gained regarding infection prevention control	5	10
	Gap between knowledge and practice	4	
	Sense of anxiety about being able to put into practice	1	
Fear for COVID-19	Reduction of the sense of fear	4	4
**Lecture on COVID-19**			
Information literacy	Obtaining accurate information	23	61
	Practicing EBM	23	
	Codifying information obtained from the media	8	
	Understanding of clinical trials	3	
	Judging of correct and incorrect information	2	
	The need for information literacy	1	
	Fluidity of information	1	
Knowledge of COVID-19	Knowledge of the vaccine	20	57
	Differences between COVID-19, SARS, and influenza	12	
	Understanding the pathology of COVID-19	11	
	Overload of information about COVID-19	5	
	Awareness of the importance of infection control	2	
	Development of therapeutic agents	2	
	Acceptance of COVID-19	1	
	Understanding of the tightening of medical care	1	
	Complications of COVID-19	1	
	Opinions from medical professionals who actually manage COVID-19	1	
	Understanding of herd immunity	1	
Educational methods	Knowledge acquisition	21	34
	Easy to understand for students	7	
	Interesting lecture	3	
	Organized information	2	
	Highly specialized	1	
Self-reflection	Awareness of one’s knowledge level and ability	10	23
	Importance of enlightening others	8	
	Recognition of the position of medical students	5	

*COVID-19* Coronavirus disease 2019, *EBM* Evidence-based medicine, *PPE* Personal protective equipment, *SARS* Severe acute respiratory syndrome

Following are some student comments from the FGI.

From comments on simulated clinical practice:

*“There were three roles, and I was able to look at each role from different perspectives. It was a good opportunity for me to practice and see that even if I think I am wearing PPE correctly, I sometimes get it wrong when I actually try it on.”*

*“It was good to know how difficult it is for medical professionals to treat COVID-19 patients that I have seen on news programs on TV. It was useful for me to have the opportunity to do it in the future.”*

From comments on the lecture:

*“Since the students are in a position between doctors and the general public, the lecture's content was based on research papers and studies and an understanding of their position. I was able to listen to the lecture comfortably without distrust.”*

*“Through the lecture, I learned the process of reading papers, checking evidence, and incorporating it into my knowledge. I thought I would do the same thing myself.”*

The inter-rater reliability results yielded a Kappa coefficient equal to 0.43. HK and CK derived categories and concepts as they emerged from data. The categories and concepts were regularly discussed with and reviewed for content by one author (KS), who has extensive experience in qualitative research, to ensure the credibility of the findings [22].

## Discussion

This study evaluated the effectiveness of COVID-19 education using simulated practice with peer role-plays and a lecture during CC. The two main findings are as follows. First, simulated practice and lectures for COVID-19 can provide a safe learning opportunity for appropriate IPC for COVID-19 and may reduce the burden due to COVID-19 patient care. Second, the simulated practice raised the students’ awareness of the burden of medical treatment on the healthcare providers and encouraged self-reflection. Moreover, the lecture also served as an opportunity to impart relevant information. The medical students were found to be highly satisfied with the medical education provided on COVID-19, and the initial objective of practicing IPC and gaining accurate information and relevant knowledge regarding COVID-19 in a safe environment was achieved.

In previous studies, on-site COVID-19 simulation concerning pediatric patients increased confidence and reduced anxiety among medical professionals [7]. Simulation of infection control practices in obstetrics using actual patients as COVID-19 cases improved compliance with standard precautions for IPC [8]. To educate students who will undertake the role of health care professionals, COVID-19 infection control simulations and peer role-playing were conducted for nursing students, and they were encouraged to learn about the use of PPE [9]. Unlike medical professionals, medical students often have insufficient knowledge and awareness about the risks of infection, including COVID-19, which may be contracted and transmitted to others, including patients in hospitals or medical centers. The lack of experience among medical personnel regarding PPE usage and inadequate IPC training led to anxiety among medical students in the UK during COVID-19 [11]. Furthermore, Harries et al. reported that adequate usage of PPE was the most important factor for medical students to feel safe resuming clinical rotations [10]. Therefore, necessary education must be provided to medical students in a safe environment. In our study, the responses to the questionnaire indicated that the burden of COVID-19 patient care was reduced. Furthermore, the FGIs revealed that students could learn about IPC, including the use of PPEs and zoning. In addition, it was suggested that realizing the burden on the healthcare providers currently engaged in COVID-19 patient care promotes self-reflection. In particular, simulation based on actual practice for COVID-19 may have contributed to a better understanding of the burden on healthcare providers. In addition, understanding the importance of collaboration among medical professions (n = 6) and learning from various perspectives (*n* = 3) in FGI may be due to the effect of the peer role-play. The peer role-plays and the simulated experience may have helped the students understand what happens in the hospital they practice under such situations.

No change in the sense of burden was observed for the situations that were not practiced in the simulation, such as responses in daily life and responses to suspected cases. Although the burden was reduced within the range of simulated situations, the students failed to apply the knowledge of IPC in other situations. Simulation with peer role-plays can be conducted even outside the hospital without any special equipment or simulated patient under appropriate settings. Further simulations may enable students to practice the actual medical treatment in a safe environment. However, an adequate supply of PPE, including N95 masks, is needed [7, 23].

The lecture also promoted self-reflection and provided relevant information on COVID-19. The students’ degree of burden in handling information was significantly reduced after the lecture. The FGIs also highlighted points such as the appraisal of information and the accuracy of information disseminated from the media. As medical information, including COVID-19, is constantly being updated, providing accurate information from the attending physicians alone is only temporary. In addition, teaching information literacy with the lecture about COVID-19 has a long-term effect on the ability to deal with infodemics. In this respect, COVID-19 can prove to be a useful topic for the students to gain information literacy because it relates to individuals’ real-life experiences and needs, and situation changes on after another.

No change was found in the fear of COVID-19 before and after our program. However, among the 44 students who were originally afraid of COVID-19 before the exercise (those who obtained scores 4 and 5), fear in 12 (27.3%) students was reduced, and in 2 (4.5%) students, an increase in fear was reported. Of the 18 students who did not originally feel afraid (Score 2), a reduction in fear was not reported, while in 10 (55.6%) students, an increase in fear was found. For students who originally had fears, the program alleviated their fear. In contrast, the students reporting a low level of fear, the ones who did not possess adequate knowledge of COVID-19, could have become more fearful after acquiring the correct information based on doctors who actually treated COVID-19 cases and evidence through our education. In addition to the fact that the FGI promoted self-reflection, our education programs were likely to help medical students find an appropriate way to deal with COVID-19 without underestimating it or being overly fearful about it. The questionnaire’s results, especially regarding fear, may have been influenced by the vaccination status of the students and the prevalence of COVID-19 in Japan. In our university, medical students were vaccinated in June 2021. However, there was no significant difference in the change in fear before and after our program, including when comparing different periods according to the pandemic.

The present study has four limitations. First, as it was carried out at just one medical school in Japan, its effects on and the comments from the participants are subject to cultural bias. Second, it was conducted in a single department, the department of respiratory medicine. Therefore, the questionnaire was administered when medical students rotated CC in respiratory medicine, which may have been influenced by education in other departments that medical students rotated to before the respiratory medicine one. Third, all participants did not have the opportunity to wear and remove PPE during the simulated practice. Finally, only self-evaluation through a survey questionnaire was conducted, and it was not verified whether the respondents adopted appropriate infection control measures.

## Conclusions

Simulated clinical practice with peer role-plays and lectures can educate medical students on the IPC of COVID-19, including wearing and removing PPE and zoning, and with respect to the reduction of the burden in taking care of the infected patients. In addition, it promotes self-reflection and a realization of the burden felt by healthcare providers and improves their information literacy on the topic. However, these effects may be limited, and repeated simulations of various situations in the care of patients with COVID-19 may be safer and more effective.

## Data Availability

The datasets generated and/or analyzed during the current study are available from the corresponding author on reasonable request. The sensitive responses of FGI from students are not publicly available because they are considered as confidential information.

## Abbreviations

CC: Clinical Clerkship
COVID-19: Coronavirus Disease 2019
FGI: Focus Group Interview
IPC: Infection Prevention Control
PPE: Personal Protective Equipment
